# Histomorphological evaluations on the frontal cortex extrapyramidal cell layer following administration of N-Acetyl cysteine in aluminum induced neurodegeneration rat model

**DOI:** 10.1007/s11011-020-00556-9

**Published:** 2020-03-24

**Authors:** Memudu Adejoke Elizabeth, Pantong Samson, Osahon Roli Itohan

**Affiliations:** 1 Department of Anatomy, Faculty of Basic Medical Sciences, College of Medical Sciences, Edo University Iyamho, KM 7 Auchi-Abuja Expressway, Iyamho, Edo State Nigeria; 2 Jiangxi University of Traditional Chinese Medicine, 818 Xingwanli Avenue, Wanli District, Nanvhang City, Jiangxi Province China; 3 Department of Anatomy, College of Medicine, Bingham University, P.M.B. 005, Karu, Nassarawa State Nigeria

**Keywords:** Aluminum, Chromatolysis, N-acetyl cysteine (NAC), Astrocytes and oxidative stress

## Abstract

Aluminum is a potent neurotoxin used in animal models of neurodegenerative diseases like Alzheimer’s disease (AD), in which oxidative stress mediates tissue pathogenesis in vivo. N-acetyl cysteine (NAC) is a glutathione precursor with reported antioxidant and neuroprotective potentials. Recent therapy for combating AD is known to provide only symptomatic relief thus necessitating the discovery of new drugs and their mechanism of action. This study was aimed to demonstrate the in vivo neuroprotective effect of NAC against aluminum (Al^3+^)-induced neuro-degeneration in rats (a model for AD). Twenty- five (25) adult male Wistar rats used for this study were divided into 5 groups: Group A = Control, B = Aluminum chloride (200 mg/kg), C = 1000 mg/kg of NAC + Aluminum chloride (200 mg/kg), D = 1000 mg/kg of NAC, E = Aluminum chloride (200 mg/kg) was orally administered daily for 3 weeks and discontinued for one week. Frontal Cortex harvested for histological analysis using Haematoxylin and Eosin stain, Cresyl Fast Violet stain for Nissl granules and Glial fibrillary acidic protein immunohistochemistry specific for astrocytes. Aluminum significantly induced oxidative stress, coupled with marked neurons necrosis, chromatolysis and gliosis in the frontal cortex, upon NAC administration, there was neuro anti-inflammatory response as seen in the significant reduction in astrocytes expression, neuronal cell death and Nissl body aggregation which attenuates neuropathological deficits induced by Al^3+^. It was shown that aluminum is a neurotoxin mediating AD-like oxidative stress, NAC has a therapeutic potential associated with its potent in vivo interaction with astrocytes in response to Al^3+^ neuro-inflammation seen in positive expression of Nissl granules and glial cells in addition to possibility of endogenous glutathione neuroprotection after withdrawal of stress mediator in neurodegeneration.

**Figure:**
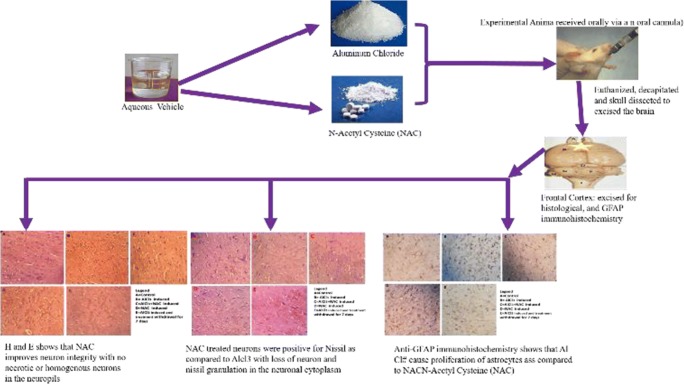
Graphical abstract

Graphical abstract

## Introduction

Neurodegenerative disorders (e.g. Alzheimer’s disease [AD]) are associated with chronic inflammation, oxidative stress (Mayeux 2010; Ahmad et al. 2018), progressive cognitive deficits and changes in neuro-behavioural pattern (Jellinger 2013). AD is linked with memory deficit and tau protein (Prema et al. 2017). There is need for new drug discovery to combat this menace as it is reported that 75% of the thirty-six million people worldwide affected with dementia are estimated to be AD patient, and it is predicated that 1 in 85 people will be affected by 2050 (Bhattacharya et al. 2014). AD is commonly seen in elderly (>65 years) but occurs in young individuals because of lifestyle, stress, depression, frustration and occupation (Reddy et al. 2017). There are drugs that clinically prove symptomatic relief for AD such as donepezil, rivastigmine, memantine, tacrine, and galanthamine (Bachurin et al. 2017). Pathological reports show that AD is characterized by deposition beta-amyloid (Aβ) plaques, reactive oxygen species (ROS), reduction in acetylcholine (ACh), hyper-phosphorylated tau proteins and glutamatergic abnormalities (Ahmad et al. 2018; Ovais et al. 2018). Aluminum was detected in senile plaques and neurofibrillary tangle in brain tissue of AD patients (McLachlan et al. 1996; Huang et al. 1997) this formed the basis for it use in animal model to study the pathophysiology of AD (Walton 2013; Virk and Eslik 2015; Ahmad et al. 2018; Chiroma et al. 2018). Aluminium exposed animals have displayed neuro fibrillary tangles formation, cholinergic neuronal axonal terminal loss, β- amyloid protein aggregation, oxidative stress and neuronal apoptosis in the hippocampus and frontal cortex site for cognition, memory and synaptic plasticity similar to the pathogenesis of AD (Praveenkumar et al. 2019). Aluminum compound gets in contact with the human system through dialysis (Yokel and McNamara 2001) drinking water and cooking utensils (Buraimoh et al. 2011) and drugs such as antacid and toothpaste (Abbasali et al. 2005). Aluminum chloride (AlCl_3_) a white salt; administered at 200 mg/kg body weight in rats and mice (Yen-Koo 1992) have demonstrated such cognitive impairments (Lide 2008; Yokel 2005).

Several therapeutic strategies, based on antioxidants agents, have been designed to prevent the impact of oxidative processes on the development and progression of AD. N-acetyl cysteine (NAC), a glutathione (GSH) precursor is currently being studied for its neuroprotective properties and mechanism of action in reversing neurodegenerative process in AD patients (Wang et al. 2013; Giancarlo et al. 2018). Mokhtari et al. (2017) described NAC as a nutritional supplement with strong antioxidant property in vivo and in vitro being a precursor for L-Cysteine capable of increasing glutathione biosynthesis. NAC prevents apoptosis in cells by increasing intracellular levels of glutathione and decreasing mitochondrial membrane depolarization (Wang et al. 2013). Glutathione, is endogenously synthesize in all mammalian cells and is currently the most studied antioxidant for treating neuropathological disorders (Shahripour et al. 2014; Giancarlo et al. 2018). Furthermore, more than 20 clinical trials have used NAC as an emerging treatment for neurodegenerative diseases or psychiatric diseases such as methamphetamine and cannabis dependence, nicotine and cocaine addiction, pathological gambling, obsessive–compulsive disorder, trichotillomania, nail biting, skin picking, schizophrenia, bipolar disorder, autism, Parkinson’s disease, multiple sclerosis, and Alzheimer’s disease- with positive effects on clinical outcomes (Samuni et al. 2013). The observed positive outcome was mediated by glutathione’s antioxidant defense role to modulate glutamatergic, neurotrophic, detoxification of electrophilic xenobiotics, modulation of redox through regulated signal transduction, storage and transport of cysteine, regulation of cell proliferation, synthesis of deoxyribonucleotide acids, regulation of immune responses, regulation of leukotriene and prostaglandin metabolism (Shahripour et al. 2014) which positively interfere with neuro- pathogenesis of AD involving neuroinflammation, glutamate neuronal activity, neurogenesis, astrocytes expression and neuronal cell death (Berk et al. 2014; Giancarlo et al. 2018). These established NAC antioxidant defense processes (Shahripour et al. 2014) have been tested in animals (Tchantchou et al. 2005) and AD patients (Adair et al. 2001) with evidence showing it potential to hinder oxidative tissue damage. Furthermore, Gil-Martínez et al. (2018) reported NAC neuroprotective strategy associated with slowing down cell death, and neuroinflammatory response related to progression of neurodegenerative disorders such as Parkinson’s disease is attributed to reduction in astroglia activity as observed in a study involving 1-methyl-4- phenyl-1,2,3,6-tetrahydropyridine (MPTP)-induced Parkinsonism model. Therefore, this study was designed to demonstrate the in vivo neuroprotective effect of glutathione precursor N-acetyl cysteine (NAC) against aluminum-induced frontal cortex neurodegeneration in rat model of AD by evaluating its effects on neuro-inflamatory mechanisms involving, astrocytes (GFAP-expression), Chromaotphilic (Nissl) bodies aggregation for protein/ neurotransmitter synthesis and neuron repair following Al^3+^ intoxication in rats.

## Materials and methods

### Experimental animals

Twenty – five (25) Adult Male Wistar rats (150–200 g) were purchased from National Veterinary Research Institute (NVRI), Vom-Jos Plateau State Nigeria. Animals were kept in well-ventilated cages and housed in the Animal House of Bingham University in standard laboratory conditions; room temperature [37 °C], the humidity of 50–60% and a 12 h dark/light cycle and access to pelleted rat fed and water ad libitum. All experiments were performed according to guidelines for the Care and Use of Animals in Research as documented in the National Research Council Guide (NRC 2011). Animals were allowed to acclimatize for two weeks before experimentation commenced.

### Aluminum chloride procurement and dose of administration

Animal studies showed that aluminum can be used as an experimental model for Alzheimer’s disease (Ali et al. 2016), it has been detected in senile plaques and neurofibrillary tangle in brain tissue of AD patients (McLachlan et al. 1996; Huang et al. 1997) hence it can exhibit neuro-oxidative tissue damage (Becaria et al. 2003; Nehru et al. 2007) similar to that seen in AD (Ramachandran et al. 2013**).** Aluminum chloride (AlCl_3_) (98%; anhydrous) was obtained from the Chemistry Department of Bingham University, Karu Nigeria. The LD_50_ for AlCl_3_ given orally is 200 –1000 mg/kg in rats and mice (WHO 1997). Our study dose was taken as 200 mg/kg as used in animal model by Yokel (2005) and Chino et al. (2005) study. In furtherance to our rationale to use 200 mg/kg, Hanaa et al. (2015) and Chiroma et al. (2018) reported it to be the best dose to model AD in animal study, as it shows the crucial pathogenesis of AD. Mode of administration was according to Auti and Kulkarni (2019) whereby, 200 mg/kg of Aluminum chloride (AlCl_3_) was given an hour before 1000 mg/kg of N- Acetyl Cysteine (NAC) for 21 days (three weeks). AlCl_3_ was dissolved in distilled water at dose of 200 mg/kg bw and given orally.

### NAC Administration procurement and dose of administration

N- Acetyl Cysteine; NAC (Swanson) 600 mg per capsule was purchased from H-Medix Abuja, Nigeria. NAC is teratogenic in rat at daily doses of 500–2000 mg/kg (Johnston et al. 1983). Although, Kasolo et al. (2019) reported that the acute oral toxicity of NAC, LD_50_ is >10,000 mg/kg bw in adult rats. AMR, (2002) reports that NAC given orally in clinical indications of 600 to 1500 mg/day but human can tolerate high doses as high as 2 to 4 g daily (AMR 2003; Gosselin et al. 2004), antioxidant potential in reducing lung tumor was reported at 1000 mg/kg (Šimkevičienė et al. 2002). Our study dose was 1000 mg/kg bw according to Dean et al. (2011) in the treatment of neurodegenerative disorders such as bipolar and schizophrenia.

### Experimental design

Rats were divided into five groups of five animals each:

- Group A: Control was orally administered normal saline for 21 days.
- Group B: Oral administration of 200 mg/kg body weight of Aluminum chloride (AlCl_3_) for 21 days.
- Group C: Oral administration of 200 mg/kg body weight of Aluminum chloride (AlCl_3_) an hour before 1000 mg/kg body weight of N- Acetyl Cysteine (NAC) for 21 days.
- Group D: Oral administration of NAC at 1000 mg/kg body weight for 21 days.
- Group E: Oral administration of 200 mg/kg body weight of Aluminum chloride (AlCl_3_) for 21 day followed by discontinuation for 7 days.

Group E was created because of the need to evaluate the possibility of the frontal cortex neurons to regenerate in the presence of endogenous glutathione following cessation of neuro- toxic assault.

### Animal euthanasia, brain tissue collection, and tissue processing

Twenty-four hours after the last administration of the study materials, the animals were euthanized and decapitated. The brains were carefully and rapidly excised, wet weight taken and then fixed in 4% paraformaldehyde in phosphate-buffered saline. The fixed brain samples were preserved for frontal cortex histological, histochemical and immunohistochemical analysis according to Bancroft and Gamble (2008) method. The frontal cortex (FC) was carefully dissected from the whole brain according to stereotaxic atlas guideline from Paxinos and Watson (2007) brain atlas. The tissues were processed using an automated tissue processor (*LEICA* TP 1050). The paraffin section was cut at 5 μm thickness and stained with Haematoxylin and Eosin (H and E) stain, Cresyl Fast Violet (CFV) stain and Glial Fibrillary Acidic Protein (GFAP) immunohistochemistry according to methods of Bancroft and Gamble (2008) and Akinrinade et al. (2015a).

### Histological staining procedure using H and E for frontal cortical neuron histo-morphology

Labelled FC slide sections were arranged in a staining slide racks, then they were immersed in two changes of xylene for five minutes each (de-waxing), then hydrated by immersing in descending grades of alcohol: 100%, 90%, and 70% for two minutes each. Then rinsed in running tap water for three minutes to wash off the alcohol. Sections were then stained with haematoxylin for five minutes, stain was differentiated in 1% acid alcohol two - three seconds; then rinsed in running tap water for three minutes for bluing to occur and counterstained in Eosin for three minutes, rinse in water and dehydrated through ascending grades of alcohol: 50%, 70%, 90% and 100% for one minute each. Slide sections were then cleared briefly in xylene set to dry in an Oven (80 °C) for sixty seconds and then covered with microscopic cover glass (22 mm X 50 mm) using DPX (Distrene Plasticizer Xylene) mountant (Bancroft and Gamble 2008; Akinrinade et al. 2015a).

### Histochemical staining Cresyl fast violet stain for Nissl granules

Labelled FC slide sections were arranged in a staining slide racks processed in the following order: 100% ethanol, 2 min; xylene, 2 min; 100% ethanol, 2 min; 70%ethanol, 2 min; distilled water, 5 min; cresyl fast violet,3 min; distilled water, two dips; 70% ethanol, 5 min; 80% ethanol, 2 min; 90% ethanol, 2 min; 95% ethanol, 2 min;100% ethanol, 5 min; xylene, 5 min; and then mounted with DPX and air-dried for microscopic observation [Bancroft and Gamble 2008; Akinrinade et al. 2015a, b].

### Glial Fibrillary Acid Protein DAB detection immunohistochemistry

Labelled FC paraffin embedded tissue sections were deparaffinized in xylene and hydrated in a descending grade of alcohol (100%, 95%, and 70%) and brought to water (distilled water). Rat anti-GFAP was prepared in PBS (Phosphate Buffer solution) 8.0 and desalted in G25 Sephadex column (protein G column); the anti-GFAP was diluted at 1:100. The GFAP antigen retrieval immunohistochemistry method using specific anti-rat polyclonal GFAP (astrocyte) was performed by incubation in citrate buffer (10 mM citric acid, pH 6.0) for 15 min and washed in PBS for 3 min. Endogenous peroxidase activity was inhibited by incubation in a protein block (1% bovine serum albumin (BSA; Sigma, Germany) and 0.3% Triton X-100 in PBS for fifteen minutes in the incubating chamber at temperature of 37 °C; to block non-specific protein reactions; slides were washed in PBS for 2 min and sections incubated in two drops of diluted primary anti-GFAP antibody (mouse GFAP- antibody Novocastra™, 1:100 dilution) and incubated for 45 min; then washed in PBS for 2 min. Thereafter, two drops of the secondary antibody (biotinylated Goat Anti-mouse IgG Novocastra dilution 1:100 dilution) was added and allowed to incubate for fifteen minutes and washed twice in PBS for thirty seconds each. The immunopositive reactions were developed using a polymer 3, 3_-diaminobenzidine tetrachloride (DAB; Sigma, Germany) with color intensification involving the use of methenamine silver kit (Sigma, Germany). The sections were counterstained in haematoxylin. Negative controls were performed by omitting the primary antibody according to the procedure described by Bancroft and Gamble (2008) and Akinrinade et al. (2015a). Sections were briefly dehydrated and cleared in xylene and air –dried. Dried sectioned were gently covered with cover slip using mounting media- DPX (Distrene Plasticizer Xylene) as described by Bancroft and Gamble (2008), Akinrinade et al. 2015a, b) and Gil-Martínez et al. (2018) methods.

### Photomicrography and statistical analysis

Photomicrographs were taken using an Olympus (Tokyo, Japan) binocular Light microscope which was connected to a 5.0-megapixel Amscope camera (Amscope Inc., Irvine, CA, USA) at 100x. The data were analyzed using one way ANOVA. Statistical significance set at *P* < 0.05. Data expressed as Mean ± Standard Deviation. Values were compared with the mean value of the control group, and mean differences were considered significant when the *p* value was less than 0.05.

## Results

### N-acetyl cysteine (NAC) improves body weight in aluminum treated animals

**Fig. 1:**
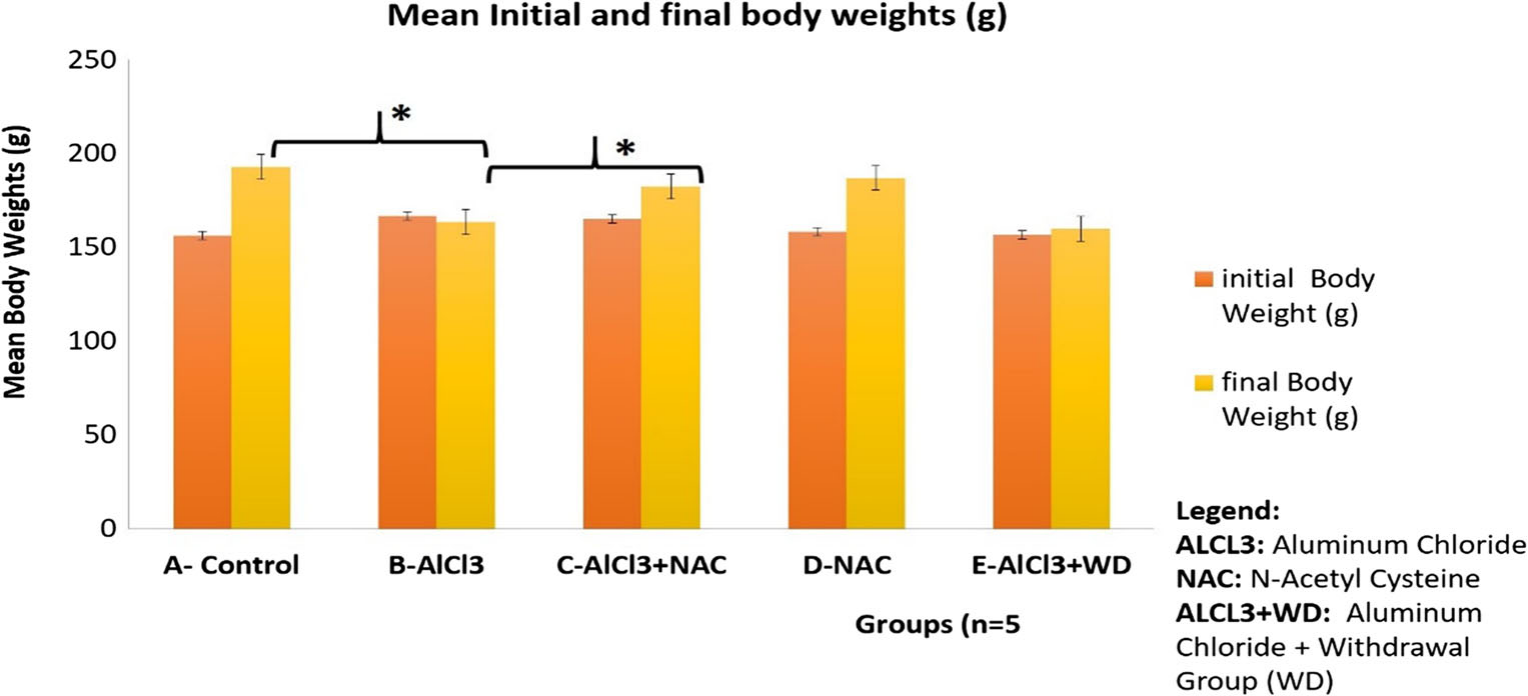
The Mean Initial and Final Body weights of Experimental Animals. Data expressed as Mean ± SD. Data Analyzed using ANOVA. Statistical Significance set at *P* < 0.05. There was no significant reduction in the initial and final body weights of group B. The final body weight decreased significantly in B* as compared to A (*p* < 0.05). Group *C had a significant increase in final body weight as compared to B and E(C* vs B and E). NAC (D) showed a significant increase in final weight gain as compared to its initial weight

The changes in body weight were done to investigate the effects of Aluminum and NAC on body weight. Our study showed that there was no significant difference in weight change in the Aluminum treated group (B) as compared to initial weight before treatment. But when comparing the final weights of Group (B) against the control (A), there was a statistically significant reduction (*p* < 0.05) (Fig. 1). NAC treated group (D) had a statistically significant increase in final body weights (p < 0.05) as compared with Group B. Group C (Aluminum Chloride +NAC) had a significance (*p* < 0.05) increase in weight gain as compared to the Aluminum Treated group (B). Group E, Aluminum Chloride post-treatment withdrawal group had no statistically significant increase in final body weight gain as compared to Group A and C.

### N-acetyl cysteine (NAC) and brain weight aluminum treated animals

**Fig. 2:**
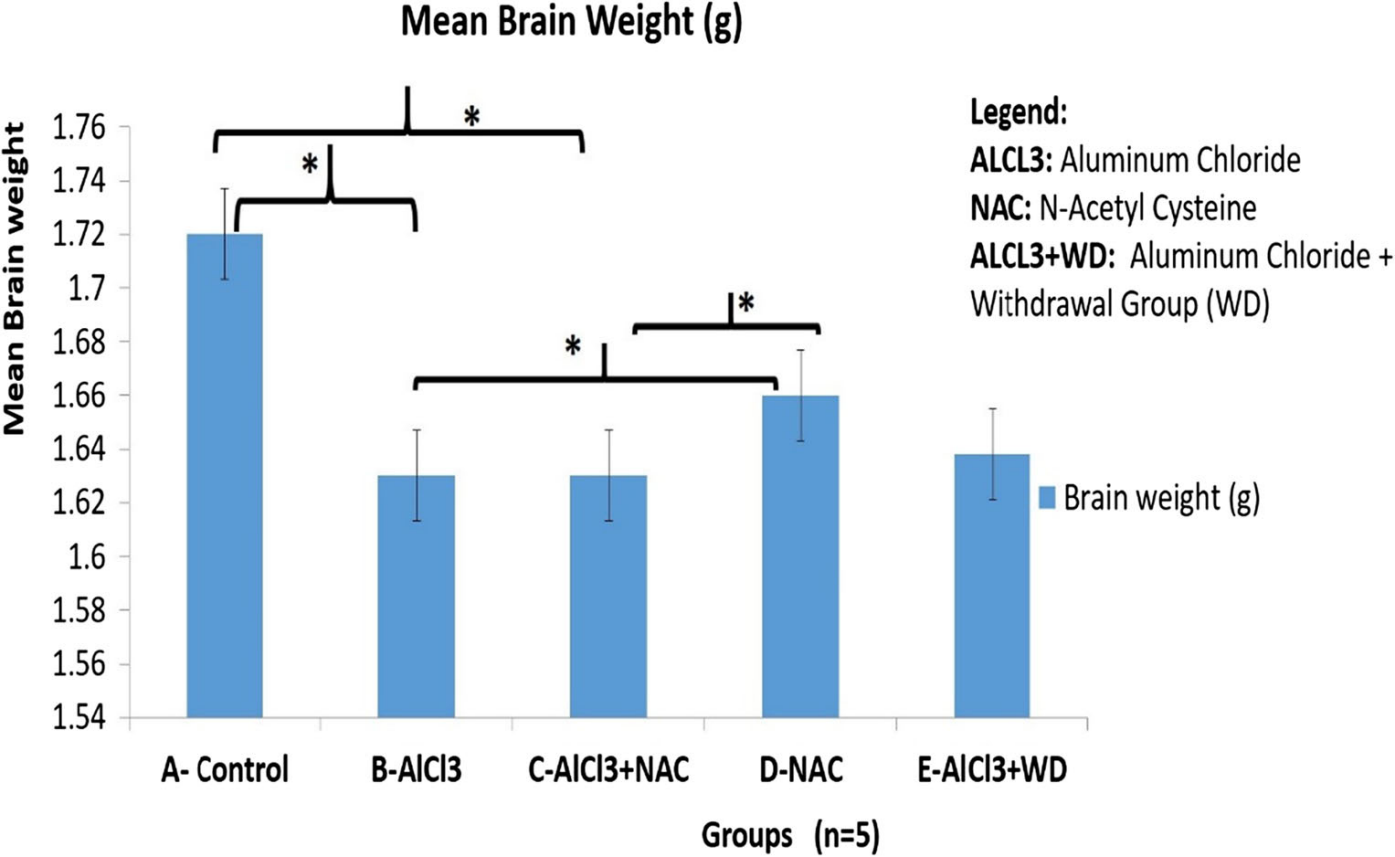
The Mean Brain weights of Experimental Animals. Data expressed as Mean ± SD. Data Analyzed using ANOVA. Statistical Significance set at *P* < 0.05. There was a statistical significant increase in A* vs B; D* had a significant increase as compared to B, at P < 0.05. There were no statistically significant difference in brain weight in B vs C at P < 0.05

In this present study, our focus to evaluate the changes in brain weight following NAC and Aluminum Chloride treated show the following observations: The brain weight of the control group A increased significantly as compared to the B- ALCl_3_ treated group (p < 0.05) (Fig. 2). NAC treated (D) had a significant increase in brain weight as compared to B at *p* < 0.05.

### N-acetyl cysteine (NAC) combats progressive neuron distortion in ALCl_3_ neurodegeneration model

**Fig. 3:**
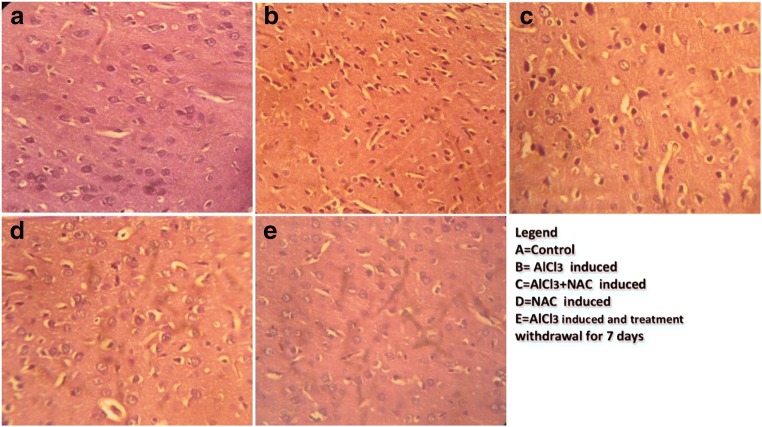
A representative Photomicrograph of the frontal cortex extrapyramidal cell layer showing the pyramidal cells of experimental Animal stained with Haematoxylin and Eosin (H and E) Stain. Control shows well-pigmented neurons and intact neuropil, Aluminum (B) treated shows severe vacuolations, necrosis and pericellular spaces around the necrotic neurons and pkynotic neurons as compared to Control (A). NAC and Aluminum + NAC posttreated shows few vacuolations and necrotic neurons in the neuropil with presence of few normal appearing neurons. The Aluminum withdrawal (Group E) shows regenerating neurons. The histomorphological appearance of Group A (Control) showed characteristically well-arranged pyramidal cells with apical and basal dendritic extensions. There are no signs of pyknosis or necrosis as compared to ALCl_3_ treatment displaying necrotic/ pyknotic cells. Comparatively, the NAC treated has normal neuron appearance as compared to ALCl_3_ plus NAC co-treated group (C) with few degenerated neurons. Group D (NAC treated) show normal Pyramidal neurons while Group E- (ALCl_3_ + 7 days post-treatment recovery group) shows self-repairing neuron, few necrotic cells and absence of vacuolation within the neuropil [Haematoxylin and Eosin (H and E) Stain. Mag. X100 Scale bar = 25 μm]

In this present study, we use Haematoxylin and Eosin (H and E) stain to demonstrate histo- architecture of the frontal cortex with specific focus on the extrapyramidal cell layer (Fig. 3).

The histological demonstration shows that the Control group (A) frontal cortex has intact neuropil with no vacuolations or pericellular spaces around the neurons. The neurons were well arranged with a central nucleus and its surrounding eosinophilic cytoplasm, as compared with the ALCl_3_ treated group (B) with a lot of necrotic neurons having homogenous cytoplasmic contents, red neurons, necrotic neurons and pericellular spaces within the neuropil. NAC treated group (D) shows normal extrapyramidal neurons and no neuronal disruption as compared to Group B. In the ALCl_3_ + NAC co-treated group (C) we observed protection of the neurons displayed by the few necrosis, vacuolation in the neuropil and presence of some healthy morphological appearance of some of the pyramidal neurons as compared with Group B. Group E shows the endogenous mechanism of neuronal restoration following the discontinuation of the assault (ALCl_3_).

### NAC restores extrapyramidal neuron integrity in aluminum induced chromatolysis and histomorphological distortion

**Fig. 4:**
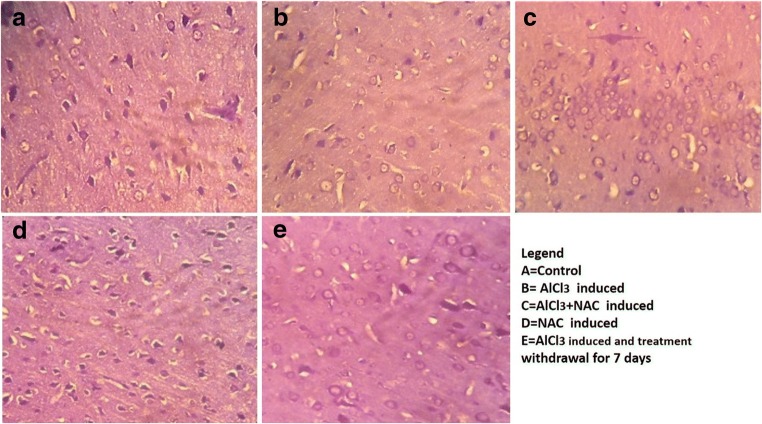
A representative photomicrograph of a section of the frontal cortex extrapyramidal cell layer stained with Cresyl fast Violet Stain. At Mag. X100. The control (A) showed Nissl positive neurons without chromatolysis. B, which is the ALCl_3_ showed the presence of chromatolysis and loss of Nissl substance. Group C (ALCl_3_ plus NAC co-treated group) showed normal neurons positive for Nissl Substance. Group D NAC treated group lack chromatolysis to has purplish stain neuron cytoplasm been positive for the presence of Nissl substance while Group E (7 days ALCl_3_ post-treatment recovery group) indicating endogenous activity of glutathione mobbing off ROS and increasing show ribosome synthesis that help repair the assaulted neuron.[Cresyl Fast Violet (CFV) Stain. Mag. X100 Scale bar = 25 μm]

Cresyl fast violet stain was used to demonstrate neuronal morphology and Nissl or Chromatophilic bodies’ aggregation use to represent ribosomes of the rough endoplasmic reticulum in the neuron cytoplasm (Fig. 4). This histochemical demonstration in our study is used to explain the interplay between ALCl_3_ and NAC on ribosome synthesis which forms the building block of amino acid in neurotransmitter requires for neurotransmission as well as neuron repair.

The frontal cortex of control and NAC (D) treated were positive for Nissl bodies stained in the perikaryon of the pyramidal neurons while Group B (ALCl_3_ treated group) stained negatively for Nissl bodies. Group C neurons were positive for Nissl bodies as compared to Group B with chromatolysis in the neuropil as compared to group E with some neuron positive to Nissl stain within the purplish neuron cytoplasm.

### NAC attenuates astrocytic cell proliferation in the aluminum induced neuroinflammation

Anti- GFAP immunohistochemistry was used to demonstrate astrocyte expression and proliferation in brain tissue. Astrocytes are supporting cells of the nervous tissue that help in neuron repair and protect against injury or neurotoxin during oxidative stress by being well expressed. This was demonstrated to evaluate the interplay between glia, neurotoxin- AlCl_3_, and NAC in the FC milieu.

Group A (Control) showed mild expression of astrocytes within the neuropil intersperse with blue stain neuronal nucleus. ALCl_3_ (Group B) treated has well expressed astrocytes as compared to A in response to neuroinflammtion - gliosis. NAC (D) also induced expression of astrocytes as compared to control but less expressed as compared to Group B- ALCl_3_, this is because there is no toxin activating cell injury that will mediate overexpression of glia cells (gliosis) as seen in Group B. However, Group C attenuated astrocytic expression as compared to Group B. Group E allowed 7 days to self-recover after 21 days of Aluminum intoxication showed a reduction in astrocytes proliferation as compared to B and D.

## Discussion

This study was done to demonstrate the in vivo neuroprotective effect of N-acetyl cysteine (NAC) by describing the interplay between aluminum-induced neurotoxicity, astrocytes neuro-inflammatory response, Nissl aggregation for protein/ neurotransmitter synthesis in neuron repair following Al^3+^ toxicity AD model. AD is characterized by oxidative stress mediated generation of ROS (Ahmad et al. 2018; Ovais et al. 2018; Ciroma et al. 2018) that leads to neuro fibrillary tangles formation, loss of neurites, reduced synaptic density, β- amyloid protein aggregation and neuronal cell death in hippocampus and frontal cortex (Praveenkumar et al. 2019).

Morphological changes in the brain and body weights were assessed to demonstrate the interplay between aluminum-induced neurotoxicity and NAC treatment. Final body weight increased in Aluminum Chloride and NAC Co-treated group as compared to B- aluminum-induced neurotoxicity model for AD. This is because NAC increased the eating pattern of the rats as compared to Aluminum treated having reduction in body weight gain due to loss of appetite as compared to the Control group (A) at *p* < 0.05 (Fig. 1). Brain volume is directly proportional to the density of the neurons, which is attributed to the thickness of the neurite and synaptogenesis in the neuropil. Also, brain volume is related to synaptic density, therefore necrosis, pruning/thinning of axon and dendrites results in reduction in synaptic density in neurodegenerative disorders like AD and this affects brain weights (Becaria et al. (2003). ALCl_3_ (B) caused a reduction in brain weight attributed to disrupted normal neuron histoarchitecture as a result of neurotoxic process leading to thinning of dendritic tree which results in reduction of brain weight (Fig. 2). KaddourTaïr et al. (2016) made a report implicating neurodegeneration being the cause of a decrease in the density of the perikaryon, trimming off of neurites or axonal outgrowth and decrease in synaptogenesis that results in decrease in brain volume. Nevertheless, NAC in Group C, caused an increase in brain weight due to the glutathione synthesized by NAC activating antioxidant mechanistic mobbing off of free radicals causing the thinning of neurite thereby preventing pruning or thinning of neurites that apparently increased the density of neuron dendrites, thereby contributing to increased synaptic density and brain weight. The ameliorative effects of NAC against Aluminum induced neurodegeneration in AD model as demonstrated in this study correlates with other studies documented by (Adair et al. 2001; Berk et al. 2014; Giancarlo et al. 2018; Gil-Martínez et al. 2018). Haematoxylin and Eosin demonstration of histological layout of the extrapyramidal layer to display histological changes between AlCl_3_ and neuron milieu interaction, shows marked necrosis, vacuolation in the neuropil and loss of neurite which affect synaptic plasticity in the extrapyramidal cell layer of the frontal cortex (Adair et al. 2001; Berk et al. 2014) as displayed in the cyto-architecture of the frontal cortex in Fig. 3B demonstrating neuronal cell death and loss of neuron histological integrity (Giancarlo et al. 2018; Praveenkumar et al. 2019). However, NAC intervention attenuates this signs in group C (Fig. 3C and 4C) by glutathione rapid mechanism of mobbing off ROS generated by Al thereby protecting against the extrapyramidal neuron cell death, degeneration of neurites and loss of Nissl bodies (chromatolysis) seen in Aluminum (Fig. 3B; 4B) (Shahripour et al. 2014; Keyes et al. 2016). Group E self-repaired because the discontinued Aluminum treatment prevent continuous generation of ROS which is up-regulated by endogenous secretion of glutathione which mobs off free radicals or reactive oxygen species already secreted. We also described the interplay between Aluminum, NAC and the rough endoplasmic reticulum of the extrapyramidal neuron by qualifying the activity of Nissl (Chromatophilic) bodies. In this study, Aluminum (B) affected the integrity of the Nissl bodies granulation in the neuron milieu (Yokel 2005) as the progression of free radicals generation seen in group B (Fig. 4B), led to disruption of cell integrity demonstrated by vacuolation within the neuropil and chromatolysis affecting the integrity of the neurons a pathophysiology of AD (Chino et al. 2005; Reddy et al. 2017) associated with oxidative stress (Asher and Guilford 2016) and loss of neuron histological integrity (Giancarlo et al. 2018, Praveenkumar et al. 2019). NAC protects pyramidal neurons from chromatolysis of Nissl bodies as seen in the increased granulation of Nissl a representation of ribosome synthesis within the neuron cytoplasm require for neuron repair and neurotransmitter synthesis (Akinrinade et al. 2015a, 2015b; Ciroma et al. 2018; Giancarlo et al. 2018 hence improving neuron appearance seen in Groups C and D as compared to Group B (Fig. 4).

**Fig. 5:**
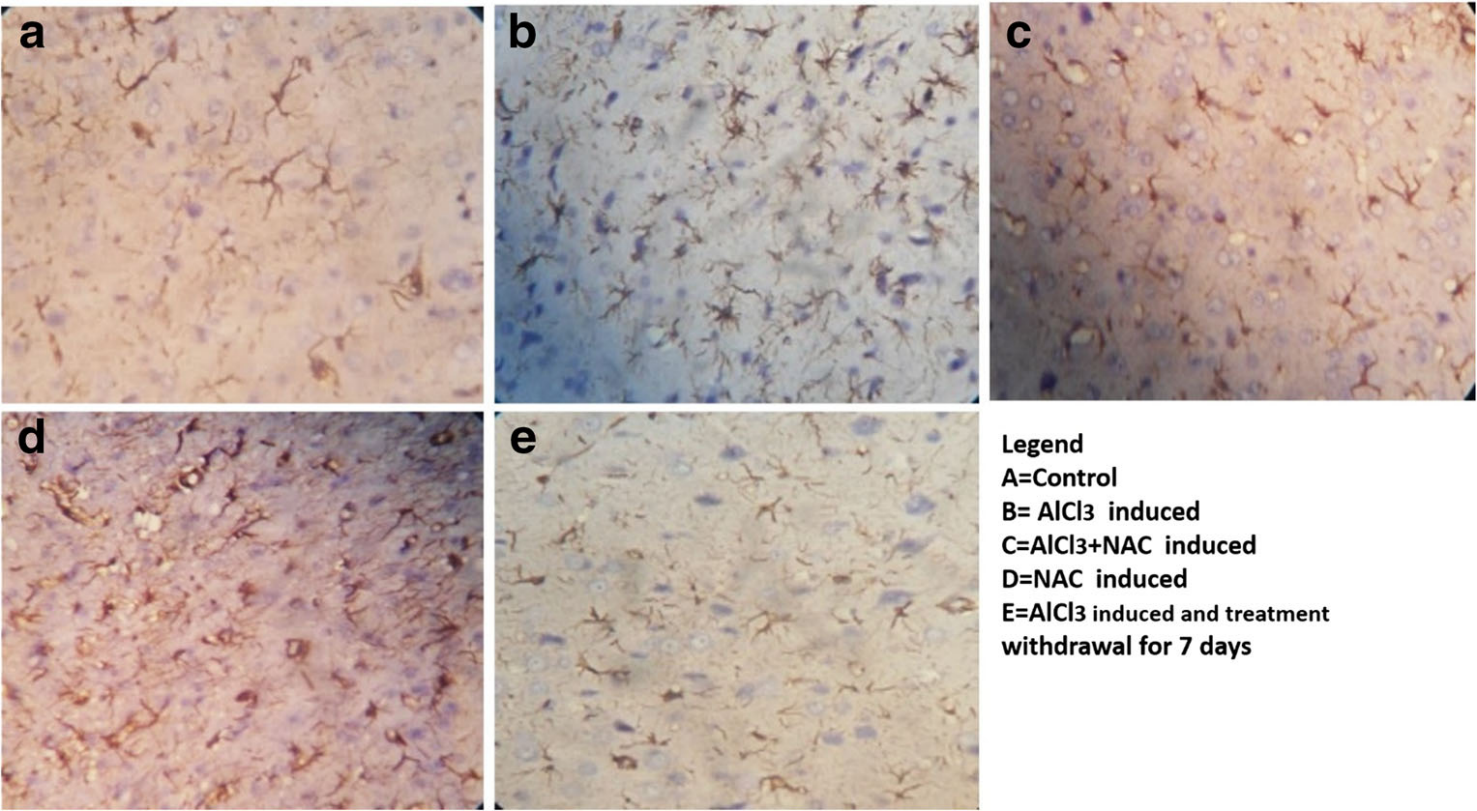
Representative Micrograph of a section of the frontal cortex extrapyramidal cell layer stained with Anti-GFAP immunohistochemical stain (treated using heat mediated antigen retrieval in citrate buffer (10 mM citric acid, pH 6.0) for 15 min). The control (A) showed a mild expression of Anti-GFAP protein with its brownish stain processes within the neuropil. Group B showed well expressed Anti-GFAP protein with an extensive cytoplasmic process demonstrated in the brownish deposition within the bluish stained neuron perikaryon. Group C and D demonstrated mild expression of the Anti-GFAP protein as compared to Group E showing extremely mild expression of astrocytes. Substance [Ant-GFAP immunohistochemistry. Mag. X100 Scale bar = 25 μm]

Aluminum caused extensive expression of astrocytes (gliosis) a condition seen in neurodegenerative conditions such as AD and PD (Raza et al. 2014; Sharhripour et al., 2014) in Aluminum treated (Fig.5B) This is attributed to a sustained inflammatory response a crucial fact for the progression of neuron loss in AD (Wang et al. 2015), this is term neuro-inflammation characterized by specialized response by activated glial cells (reactive gliosis) an oxidative stress mechanism (McGeer and McGeer 2008; Miller et al. 2009). According to Deepmala et al. (2015) and Batlle et al. (2015) astrocytes are active during neuroinflammation to help neuron repair, support it during injury or toxic assault and restore cerebral homeostasis. However, N-acetylcysteine (NAC) in vivo treatment due to glutathione synthesis, feds glutathione into the neuron milieu thereby regulating redox activating mechanism leading to a reduction in astrocytes expression seen in Fig. 5C and D as compared to 5B (Keyes et al. 2016; Ortiz et al. 2016). Thereby implicating NAC- glutathione potential to significantly decrease glial response and neuronal cell death (Giancarlo et al. 2018). The self-recovery group (Group E) was to access the process of endogenous glutathione generation that will reverse neuroinflammatory mediated gliosis following withdrawal of the neurotoxic compound. We observed that seven days after Aluminum treatment cessation, the extrapyramidal neurons were able to endogenously produce glutathione reported to be ubiquitously synthesized in all mammalian cells (Shahripour et al. 2014) as compared with Group B, this endogenous glutathione, mediates neuron repair by mobbing off ROS and up regulating brain energy metabolism to sustain the stressed neuron repair process. Activation of glial cells may reduce or delay the progression of neurodegeneration (Du et al. 2016; Durieux et al. 2015) thereby ameliorating pathological features associated with H_2_O_2_-mediated neuronal toxicity and provided protection against apoptotic cell death (Gao et al. 2017) due to Aluminum intoxication. It was established that aluminum activated oxidative stress can deplete the cellular level of glutathione (Asher and Guilford 2016) associated with chromatolysis and gliosis seen in Fig. 3B; 4B and 5B which are common pathologic features of neurodegenerative disorders such as AD (Gu et al. 2015; Homma and Fujii 2015). Also NAC antioxidant protective mechanisms increased concentration of glutathione needed for the conjugation of NAPQI hence acting as an anti-inflammatory and antioxidant agents (Farrell 2018) was also established.

## Conclusion

The present study showed that, NAC has neuroprotective effect in aluminum chloride induced neurotoxicity linked to neuron cell death, chromatolysis and gliosis thereby increasing endogenous glutathione to combat stress mediated oxidative tissue damage. This effect of NAC interplay within the neuron milieu, with astrocytes and ribosomes may be use as its mechanism of action to manage Alzheimer’s disease.

### Main points

- NAC positive interference in neurodegenerative conditions such as AD, was demonstrated in its extensive synergistic mechanism of action along with astrocytes in a neurotoxic (Aluminum) milieu in vivo by activating neuroinflammatory response leading to:
  - A decrease in glial expression and,
  - A significant decrease in necrosis and chromatolysis via this mechanisms

An indirect antioxidant effect of NAC being a precursor of Cysteine the rate limiting factor for glutathione synthesis is to replenish the reduced neuron ubiquitous/ endogenous glutathione (GSH).

b) **The significance of endogenous** glutathione production is to repair neurons injured during exposure to oxidative stress mediator caused due to stress, lifestyle or occupation in AD patient shows neuron can self- repair over time following changes removal of such stress mediators
